# Monitoring biosecurity in poultry production: an overview of databases reporting biosecurity compliance from seven European countries

**DOI:** 10.3389/fvets.2023.1231377

**Published:** 2023-08-15

**Authors:** Mattias Delpont, Luis G. Salazar, Jeroen Dewulf, Artur Zbikowski, Piotr Szeleszczuk, Anne-Christine Dufay-Lefort, Nathalie Rousset, Annick Spaans, Arthi Amalraj, Giuditta Tilli, Alessandra Piccirillo, Aitor Devesa, Sandra Sevilla-Navarro, Hilde van Meirhaege, László Kovács, Ákos Bernard Jóźwiak, Jean-Luc Guérin, Mathilde C. Paul

**Affiliations:** ^1^IHAP, Université de Toulouse, INRAE, ENVT, Toulouse, France; ^2^Department of Internal Medicine, Reproduction and Population Medicine, Faculty of Veterinary Medicine, Ghent University, Merelbeke, Belgium; ^3^Department of Pathology and Veterinary Diagnostics, Institute of Veterinary Medicine, Warsaw University of Life Sciences, Warsaw, Poland; ^4^ITAVI, Institut Technique de l'Aviculture, Pisciculture et Cuniculture, Paris, France; ^5^Southern Agriculture and Horticulture Organization, Hertogenbosch, Netherlands; ^6^Department of Comparative Biomedicine and Food Science, University of Padova, Legnaro, Italy; ^7^Centro de Calidad Avícola y Alimentación Animal de la Comunidad Valenciana (CECAV), Castellón, Spain; ^8^Vetworks BV, Aalter, Belgium; ^9^Department of Animal Hygiene, Herd Health and Mobile Clinic, University of Veterinary Medicine, Budapest, Hungary; ^10^Digital Food Chain Education, Research, Development and Innovation Institute, University of Veterinary Medicine, Budapest, Hungary

**Keywords:** prevention, surveillance, audits, avian influenza, broilers, egg layers, turkeys

## Abstract

Compliance with required on-farm biosecurity practices reduces the risk of contamination and spread of zoonotic and economically important diseases. With repeating avian influenza epidemics in the poultry industry, the need to monitor and improve the overall level of biosecurity is increasing. In practice, biosecurity compliance is assessed by various actors (e.g., academic, private and public institutions), and the results of such assessments may be recorded and gathered in databases which are seldom shared or thoroughly analyzed. This study aimed to provide an inventory of databases related to the assessment of biosecurity in poultry farms in seven major poultry-producing European countries to highlight challenges and opportunities associated with biosecurity data collection, sharing, and use. The institutions in charge of these databases were contacted and interviewed using a structured questionnaire to gather information on the main characteristics of the databases and the context of their implementation. A total of 20 databases were identified, covering the gamut of poultry species and production types. Most databases were linked to veterinary health authorities or academia, and to a lesser extent interbranch organizations. Depending on the institutions in charge, the databases serve various purposes, from providing advice to enforcing regulations. The quality of the biosecurity data collected is believed to be quite reliable, as biosecurity is mostly assessed by trained farm advisors or official veterinarians and during a farm visit. Some of the databases are difficult to analyze and/or do not offer information concerning which biosecurity measures are most or least respected. Moreover, some key biosecurity practices are sometimes absent from certain databases. Although the databases serve a variety of purposes and cover different production types, each with specific biosecurity features, their analysis should help to improve the surveillance of biosecurity in the poultry sector and provide evidence on the benefits of biosecurity.

## 1. Introduction

Biosecurity practices are a means to prevent the introduction, spread, and persistence of pathogens in livestock production. As pathogens can be transmitted via numerous, direct and/or indirect transmission routes, a range of biosecurity measures need to be implemented at the farm level (1, 2). These measures include day-to-day routine practices (e.g., using farm-specific clothing and equipment), the design of adapted premises (e.g., building layout which allows the separation of zones with different sanitary statuses), and the implementation of controls (e.g., monitoring the efficacy of cleaning and disinfection) (3).

To provide efficient protection, biosecurity measures should be applied continuously. Although biosecurity guidelines are available in a wide range of contexts, studies conducted in various countries, and various animal production systems have revealed that some guidelines are rarely followed (4–6).

To verify whether and how biosecurity measures are applied, assessments of on-farm biosecurity compliance are often based on checklists (7). In some cases, the analysis of the checklists may consider the relative importance of different biosecurity measures, and then propose a biosecurity score for a given farm (8–10). Biosecurity assessment tools may serve various purposes. They can be used to control and enforce the implementation of biosecurity regulations (11, 12) or rules, including those for certification purposes (13). They also can be used to provide advice to farmers. The advice can be provided by a farm advisor (e.g., farm veterinarian in the context of a disease prevention plan) or by farmers themselves when the tool is meant for self-assessment. Finally, biosecurity may be assessed for research purposes, such as identifying the risk factors for a given disease and proposing targeted intervention strategies (14, 15). Due to the variety of reasons prompting the collection of biosecurity data, some farms may have their biosecurity practices assessed several times over a short period of time.

In poultry production, biosecurity measures have been the focus of considerable and increasing attention to prevent severe and highly transmissible diseases (e.g., avian influenza, Newcastle disease, and infectious laryngotracheitis) (16, 17) and food-borne zoonotic diseases (e.g., *Salmonella* spp. or *Campylobacter* spp.) (18, 19).

Documenting the characteristics and uses of existing biosecurity assessment tools and increasing access to biosecurity compliance information could serve several purposes. These include (1) enabling users to avoid collecting data that already is available, (2) identifying common or specific difficulties, and (3) finding efficient ways to overcome them.

In this paper, we aimed to identify the challenges and opportunities of on-farm biosecurity data collection. We focused on the context of biosecurity assessments (reliability, exhaustivity, and coverage), data sharing, and data use. An overview of existing poultry biosecurity compliance databases identified in seven European countries participating in a European thematic network on biosecurity provided the basis for our study.

## 2. Materials and methods

To identify poultry biosecurity databases, correspondents in each of the seven European countries involved in the NetPoulSafe consortium (20) were contacted. These correspondents then identified stakeholders who managed or owned poultry biosecurity databases and interviewed them using the questionnaire provided by this study’s authors. The questionnaires collected information on the characteristics of each database (one questionnaire was filled in per database). The questionnaires were completed between May 2021 and February 2022.

A database was considered as any source of biosecurity data (digital or physical) obtained using a given questionnaire, in a given context, by a given organization (private or public). Although a database is supposed to gather data in a single place or file, biosecurity assessment systems in which data were collected but not gathered and assembled also were included. As the aim of the study was to describe active or “recent” systems, it was decided to include databases with data produced between May 2011 and May 2021.

The questionnaire used in this study aimed to gather information describing the context and the content of the databases. It included five parts: (1) the number of farms, the type of production, and the sampling procedures (13 questions), (2) data ownership, storing and sharing conditions (eight questions), (3) the aims of data collection and the intended feedback (three questions), (4) how the data were obtained (two questions), and (5) the types of biosecurity practices which were assessed (21 questions). The questionnaire mainly contained closed questions, but spaces for comments were left periodically so that additional information could be recorded when closed questions would not suffice. The questionnaire is accessible in Supplementary Table 1.

The questionnaires were filled in using an online form, created with “Sphinx iQ 2” software (21), and the results were stored on a secure online server. The results were analyzed with R software (22).

## 3. Results

A total of 20 databases with information on biosecurity compliance on poultry farms were identified in the seven participating countries. One database, Biocheck, created by Ghent University (Belgium), provided information on three different participating countries (Belgium, Spain, and the Netherlands). France reported seven databases, Poland reported four databases, Spain, Belgium, and Italy reported two databases each and Hungary and the Netherlands one database each.

### 3.1. Types of farms and production stages

The biosecurity compliance databases covered various poultry species and types of production. Broiler chickens were included in 16 databases, egg layers in 14 databases, turkeys in 14 databases, ducks in 11 databases, and others types of poultry (e.g., guinea fowl and game birds) in eight databases. The databases also covered various production stages: final production in 19 databases, selection and multiplication (i.e., all stages involved in reproduction) in 10 databases, and hatcheries in five databases.

### 3.2. Database owners and aims

**Figure 1:**
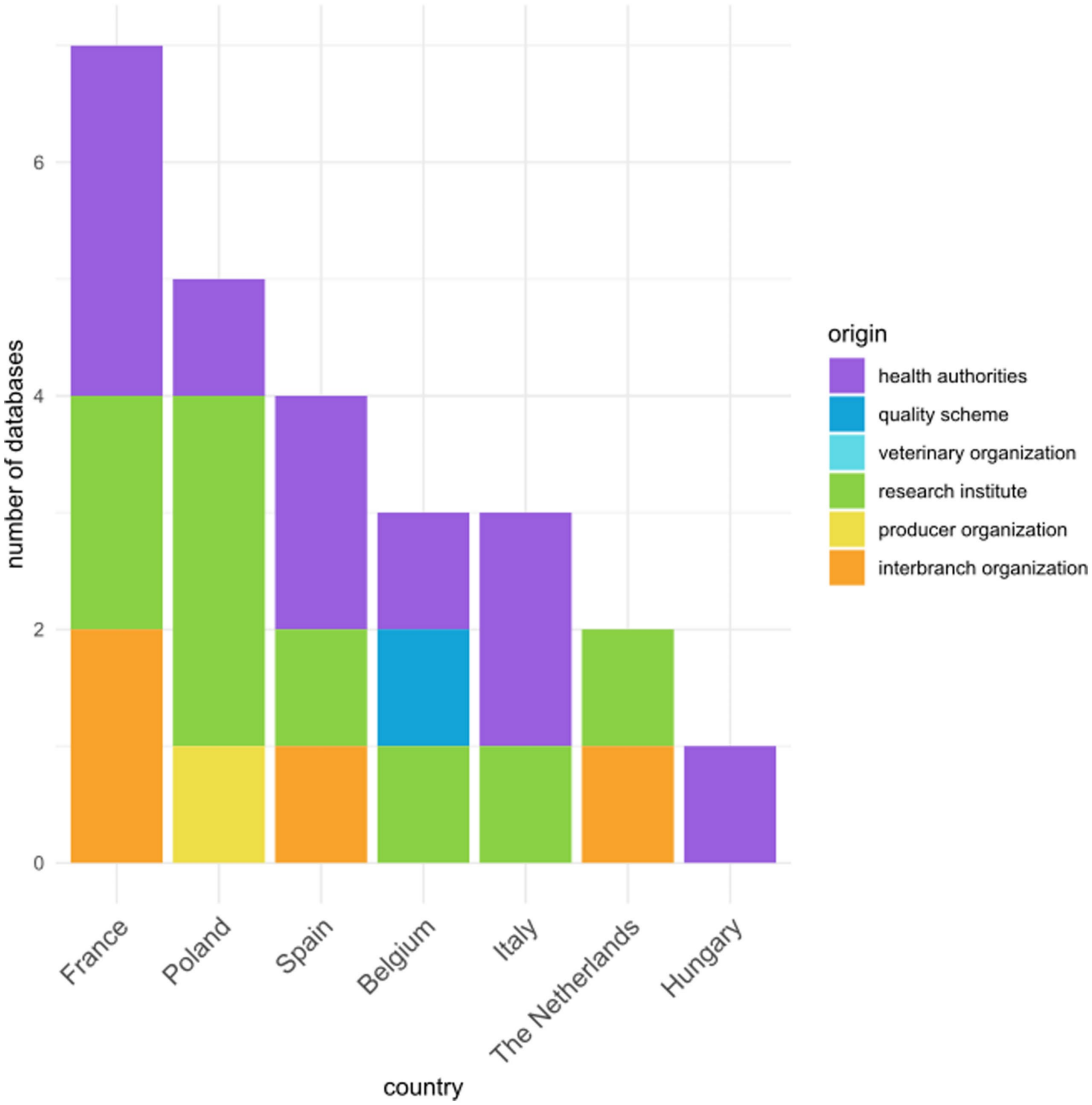
Origin of the 20 referenced poultry biosecurity compliance databases, in each of the seven participating countries. When a database involved several countries, it was counted once for each country.

The most common origin for a database was veterinary health authorities (*n* = 10), followed by research institutes (*n* = 9) and interbranch organizations (*n* = 4). Interbranch organizations are defined as organizations gathering actors involved in the production chain of a product. For example, in a given country, the interbranch organization for broiler chicken includes representatives from different producer organizations (also called integrators), syndicates, hatcheries, breeder stock and genetics companies, feed companies, slaughterhouses, and distributors. A producer organization and a quality scheme were also at the origin of one database. No veterinary organization was at the origin of any database. The origin of the databases is presented by country in Figure 1.

**Figure 2:**
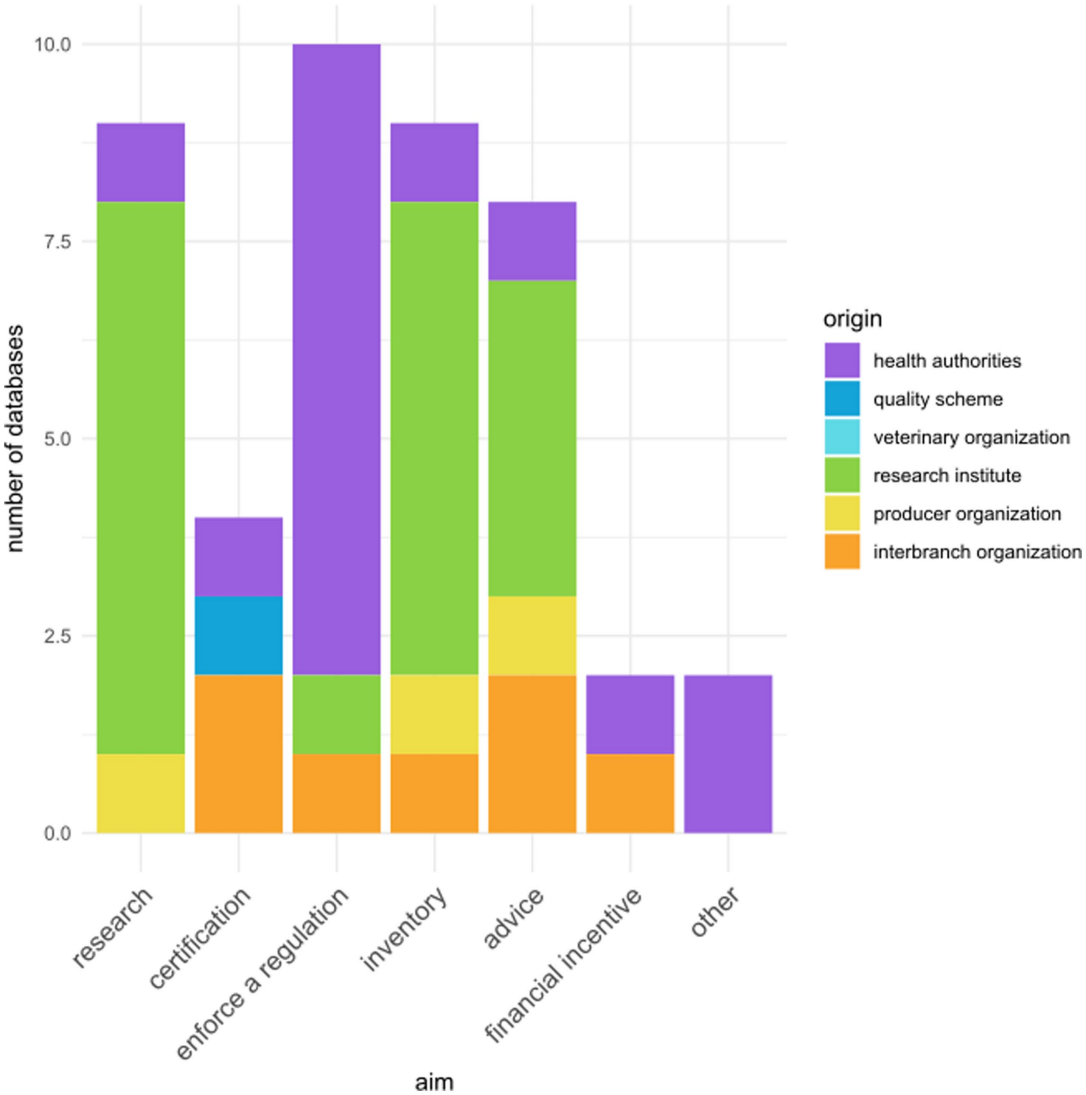
Aim of the 20 referenced poultry biosecurity compliance databases, in relation to their origin. Some databases had multiple origins or aims, therefore all combinations were accounted for.

The aims of data collection were the following: enforce a regulation (*n* = 8), provide an inventory (i.e., an overview of current practices; *n* = 8), give advice to farmers (*n* = 7), research (*n* = 7), provide a certification (*n* = 4), serve as a condition to give an incentive to farmers (*n* = 2), inform risk analysis (*n* = 1), and sensitize farmers to the importance of biosecurity (*n* = 1). The relation between aim and origin is given in Figure 2.

Among the eight databases related to the enforcement of a regulation, some were specifically dedicated to the control of a given pathogen (two on avian influenza and two on *Salmonella*) and some other were not specific to any given pathogen, although set in a context of avian influenza epidemics.

### 3.3. Participation and coverage

The participation and the coverage of the poultry farms targeted were related to the origin of the databases. Different situations consequently were identified. The first consisted of mandatory annual inspections targeting 100% of the study population carried out under veterinary health services inspection programs. The second also involved mandatory annual inspections carried out by veterinary health services, but these only targeted a fraction (estimated to be below 30%) of the total number of poultry farms due to their high numbers. The third situation consisted of a non-mandatory sanitary certification program managed by veterinary health services which granted farmers additional compensation if a mandatory culling protocol for *Salmonella* control was implemented. The fourth consisted of an industry initiative (quality label or interbranch organization) with mandatory or strongly advised participation and a very important coverage of the targeted population (70–90%). The fifth situation consisted of research protocols, with a non-mandatory participation and a lower coverage of the poultry industry or target population (lower than 30%). A summary of the different situations regarding participation and coverage is given in Table 1, along with the number of countries concerned.

**Table 1:** Participation and coverage of the 20 referenced poultry biosecurity compliance databases.

Origin	Participation	Estimated coverage of the national poultry industry or targeted farm type (%)	Number of biosecurity assessments (audits, interviews, etc.)	Number of countries concerned
Veterinary health services	Mandatory	100	[145–15,247]^*^	4^**^
Veterinary health services	Mandatory	[20–30]	[350–2,914]	2
Veterinary health services	Voluntary	Not provided	Not provided	1
Industry (Interbranch organization or quality label)	Strongly advised or mandatory	[70–90]	2,000^*^	3
Research institute	Voluntary	≤20^***^	[30-350]	6

^*^Data available for only two databases.

^**^Includes only countries with information on coverage.

^***^No lower interval given because of lack of information for coverage, which was estimated as very low.

### 3.4. Conditions of biosecurity assessment

The conditions of biosecurity assessment refer to the type of person involved in data collection, the items included in the checklist, and the way the data were collected (postal, telephone or face-to-face interview, and/or inspection of farm documents and premises). In most cases, data were collected during farm visits (*n* = 18) and the people most commonly involved in data collection were farm veterinarians (*n* = 7) and public health authority veterinarians or technicians (*n* = 6). The complete results for the conditions of data collection are presented in Table 2.

**Table 2:** Conditions of data collection in the 20 referenced poultry biosecurity compliance databases.

Conditions of data collection		Number of databases concerned
How was biosecurity assessed?		
	Farm visit	18
	Face-to-face interview	10
	Online questionnaire	2
	Phone interview	1
	Postal questionnaire	1
Who assessed biosecurity?		
	Farm veterinarian	7
	Public health authority veterinarian or technician	6
	External auditor	4
	Researcher	4
	Technician	4
	Farmer	3

In some databases, biosecurity could be assessed by different types of people and by different means.

### 3.5. Format and feedback

Half of the databases (*n* = 10) were not structured in a single file. Among these, two databases were not digitalized and the data, only stored on paper, were sometimes stored in different places. In general, data were first collected on paper (*n* = 13) and then digitalized, individually or gathered in a single file.

Providing feedback to farmers was considered to be done by 16 databases. In most cases, the feedback consisted of an individual report given to the farmer (*n* = 15), sometimes containing a progress plan (*n* = 2) or providing a benchmark for the farmer (*n* = 2). In some research projects, the projects’ overall results also were given to the farmers (*n* = 5) or published in a public report (such reports could not be quantified, but were produced in at least one case) or in a scientific publication (when data were collected for scientific purposes).

### 3.6. Database accessibility by other institutions

**Figure 3:**
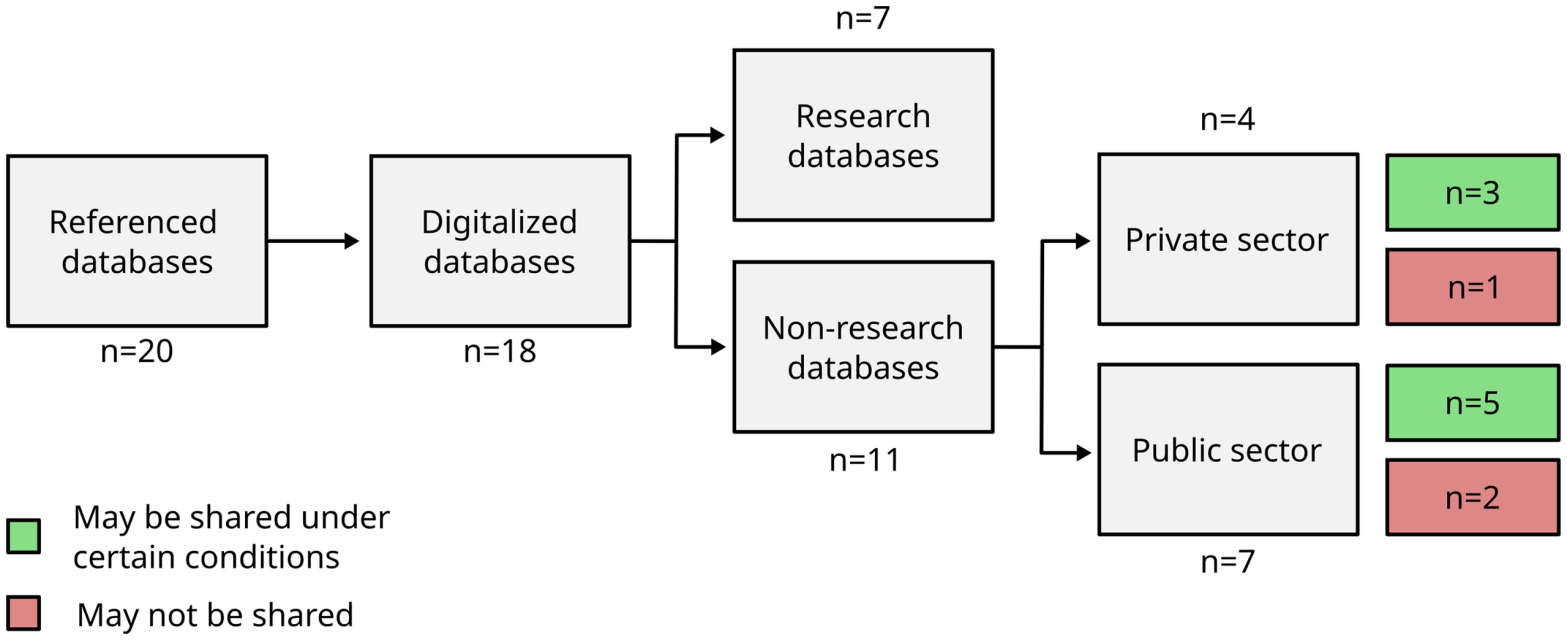
Overview of the possibility to access and analyze the 20 documented biosecurity databases by other organizations. Research databases are not detailed here.

Among the 18 databases existing in digital format, seven were associated with a research project. Among the 11 non-research databases, four were owned by private sector organizations, while seven were owned by public sector organizations. Again among these 11 non-research databases, eight could be shared under certain conditions, according to their owners. In this case, data sharing refers to obtaining non-processed data on biosecurity assessment. One of the database owners declared that when data were collected on biosecurity, it was specifically agreed with the farmers that the data could not be shared. The conditions for data sharing were to establish a specific agreement between the owner and the institution in charge of the analysis. Some specific requirements also were set with regard to the choice of the institution in charge of the analysis, for example, public health services only, or public institutions from the same country only. In addition to that, most requirements for data sharing included a step of anonymization and/or the removal of any personal data. Finally, when the eight database owners (those who declared that they could share their databases under certain conditions) were asked to share some parts of their databases with the NetPoulSafe project, two of them eventually refused. The reason given was that if poultry industries from different countries were compared, there could be a negative economic impact (in terms of international trade). The overview of the number and type of non-research databases accessible for analysis by another institution are given in Figure 3.

### 3.7. Biosecurity aspects covered by the databases

**Figure 4:**
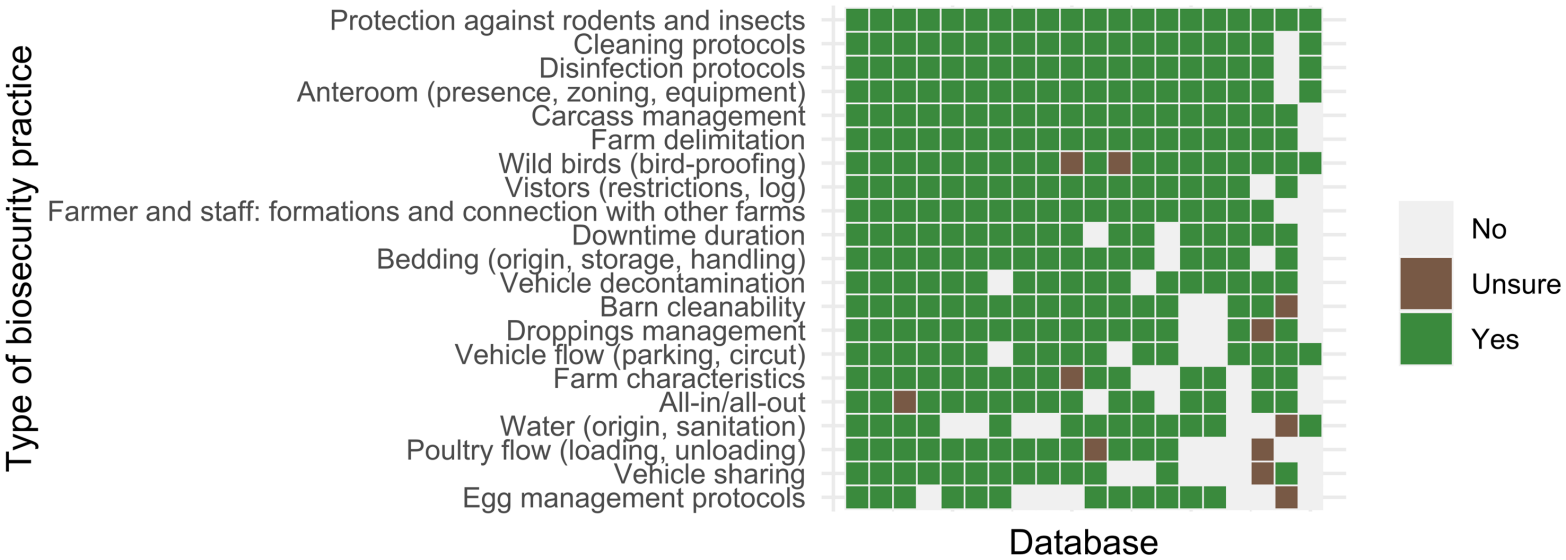
Types of biosecurity measures included in the biosecurity assessment checklists related to the 20 poultry biosecurity compliance databases. Green tiles indicate the presence of at least one checklist item from a given type of biosecurity in a given database.

The types of biosecurity practices which were included most frequently in the databases comprised vermin management (20/20), cleaning and disinfection protocols (19/20), anteroom presence and equipment (19/20), carcass management (19/20), and farm delimitation (19/20). The types of biosecurity practices which were included least frequently were egg management procedures (12/20), water quality (13/20), poultry flow management (13/20), equipment sharing between farms (13/20), description of the farm characteristics (15/20), farm management in all-in/all-out or multiple-age system (15/20), and vehicle flow management (16/20). The main types of biosecurity practices covered by the different databases are given in Figure 4.

## 4. Discussion

The originality of this study is to draw from experiences of biosecurity data collection systems in a variety of European countries to identify challenges and opportunities, and provide recommendations for further improvements.

This study has some limitations related to the identification of databases. It is possible that this study overlooked some poultry-related databases when the databases or their owners were unknown to the investigators at the country level. In particular, some academic research databases with (still) unpublished results may have been missed. Furthermore, in countries where the management of biosecurity is operated at the provincial or regional level rather than the national level, some initiatives may have been missed, given the multiplicity of potential contact people. However, in such cases, the results may be extrapolated to other zones. There were only seven European countries involved in this study, but with some exceptions (e.g., Germany), the most important European poultry-producing countries were included. It would be interesting to analyze other European countries (and also important non-European poultry-producing countries) and farmed species in further research. Another limitation to the study was the difficulty of assessing with precision, for each database and then for each region or country, the percentage of poultry farms included. On the one hand, the coverage for research-associated databases was low (23, 24) for both practical reasons and because they did not necessarily require high coverage. In databases fed by auto-evaluation tools, the number of farms covered also can vary greatly depending on participants’ willingness to use the tools and whether the tools are user-friendly (25). Moreover, in such databases, because assessments are anonymous, it may be not be possible to know if several assessments were produced on the same farm. On the other hand, databases associated with official inspection programs presented for each country or region cover an important share of the poultry production.

In this study, veterinary health services emerge as the main actors in biosecurity assessment. However, we found that the biosecurity assessment systems used by veterinary health services vary greatly. The other types of organizations involved in biosecurity assessment also are highly diverse. One of the main aims of the reported databases was the enforcement of biosecurity regulations. Therefore, most countries had at least one biosecurity assessment system managed by official veterinary services. The only exception was the Netherlands, where the main biosecurity assessment system is managed by an interbranch organization. Nevertheless, it covers a very important share of the poultry farms in the country. Interestingly, in some countries, the official veterinary health services may manage several biosecurity databases. In France, for instance, diverse biosecurity assessment systems were developed in response to the emergence of specific pathogens over time; the oldest system is linked to a *Salmonella* control plan, whereas the newest is linked to a plan to control the more recent highly pathogenic avian influenza (HPAI) epidemics. Furthermore, biosecurity databases managed by veterinary health service may also serve purposes other than the enforcement of biosecurity regulations. For example, when a *Salmonella* outbreak takes places on a farm that has been shown to meet biosecurity requirements, the farmer obtains a larger financial compensation. Similarly, in Belgium and Germany, financial compensations for HPAI outbreaks in poultry farms currently depend on the level of on-farm biosecurity (26, 27). Surprisingly, giving advice to farmers can also be the aim of a biosecurity monitoring system managed by official veterinary health services. In France, in order to provide advice to farmers, some mandatory visits involving a biosecurity assessment are conducted by farm veterinarians and funded by the veterinary services (12). For certification purposes, biosecurity assessment was sometimes included in a broader framework, in addition to other quality-related aspects, such as animal welfare or traceability (three different databases, described in Belgium and France). In the landscape of poultry biosecurity databases presented in this study, the Biocheck database presents some unique features in terms of objectives and origin. It consists of an online self-assessment tool which also records the biosecurity data entered by the end-user. Since this tool was developed by researchers, the data obtained and the tool itself are regularly used for research purposes worldwide.

This study aimed to identify the challenges and opportunities associated with biosecurity assessment systems and their databases. This can be considered at two levels: database construction (conditions of data collection) and data use (sharing, analysis).

To begin with, at the level of data collection, we expected the quality of the data to be heterogeneous. One reason for this is that the person conducting a biosecurity assessment is known to play a role in the reliability of the information obtained (7). In many of the databases described, biosecurity was assessed by a trained person (other than the farmer) and following a standardized procedure. Moreover, in many cases, farms visits were conducted, allowing the use of farm observations and written documents, adding to the reliability of the data obtained. However, some of the databases relied on data collected through self-assessments, which may be biased. A second reason is that we found, with regard to the overall content of biosecurity assessment checklists that some key aspects of farm biosecurity were at times missing. For example, checklist items on water quality and sanitation were absent in 8/20 databases, despite the importance of water in fecal-oral contaminations (28, 29). Checklist items on vehicle sharing also were absent in 7/20 databases, despite the known risk of farm-to-farm contamination during avian influenza and infectious laryngotracheitis epidemics (17, 30). The same remarks apply to the conditions of poultry unloading (31), the absence of an all-in/all-out system (32, 33), and vehicle flow management (33, 34). In this study, some aspects of on-farm biosecurity were described as being present in most databases, but this does not mean that they were assessed in a reliable manner. For example, carcass management, assessed in 19/20 databases, may rely solely on asking farmers whether they pick up carcasses daily (35) rather than addressing the conditions under which the carcasses are brought to the storage room (36). Collecting and analyzing the original checklists would help identify more precisely which biosecurity items may be missing from biosecurity assessment systems. However, we need to keep in mind that some differences in biosecurity checklists were to be expected due to the specific characteristics of certain production stages. Egg management procedures, reported in 12/20 databases, are obviously specific to egg-layer production systems (including poultry breeders). With regard to the conditions of data collection, we should keep in mind that repeating similar biosecurity assessments on farms may confuse or annoy farmers. In addition, the cost associated with unnecessary campaigns of on-farm biosecurity assessment must be considered for various stakeholders. In France, for poultry farms, the veterinary authorities are now validating biosecurity assessments conducted in the framework of industry certifications and controls. In Ireland, the veterinary authorities also fund free biosecurity assessments once a year for poultry and pig farmers, using a checklist of academic origin. The last part of database construction concerns entering the biosecurity assessment data in the database, that is to say digitally. While the people in charge of biosecurity assessment may find it more convenient to collect data on paper, it takes additional time to input the data into the database. In two cases, biosecurity data were collected but not entered into any database.

At the level of database access and analysis, several aspects require discussion. First, this study highlights the challenges associated with data sharing on biosecurity assessment. Some reasons are linked to database owners’ strategies and prospects, or relations and contracts between organizations. Sharing biosecurity databases offers opportunities to implement different types of analysis strategies, ranging from transversal analysis across different countries to connection with other types of data (health, production, etc.) in order to document the relationship between biosecurity characteristics and various outcomes. Sharing and connecting biosecurity compliance databases offers the possibility to compare biosecurity practices between different poultry production species, farming systems, and countries. However, such comparisons must be interpreted cautiously. The protocols for biosecurity assessment are heterogeneous (with regard to sampling, reliability of compliance-assessment, the choices to include or not some biosecurity items and their precision) and the local epidemiologic and economic context does not require the same biosecurity standards to be followed (37). For example, poultry breeder farms, in which animals have a great economic value (the economic consequences of an infection would then be more important) or farms located in a high-risk area (the probability of farm infection would be higher) would require stricter biosecurity measures. Similarly, local regulations may differ strongly. For example, rendering is the only authorized way to dispose of poultry carcasses in Europe while on-farm composting and incineration is allowed in North America. Nevertheless, a comparison of biosecurity compliance databases could provide—to a certain extent–a useful benchmarking tool. In addition, it could be used to highlight which practices may be more easily adopted regardless of the context. Similarly, it could help identify common challenges and the measures that have been successful in addressing them, providing lessons learned to benefit others. Another key aspect of biosecurity database access and analysis is the possibility to produce knowledge on the benefits of biosecurity. The main challenge here lies in allowing research organizations to use the databases. One of the main barriers cited by farmers for the implementation of biosecurity measures is the lack of available evidence on the benefits of biosecurity on animal health, antibiotic use and animal productivity (38–40). Previous works have used existing biosecurity databases to prove the benefits of biosecurity. For example, a study pointed out the main biosecurity risk factors associated with avian influenza infection in poultry farms, using a database of biosecurity inspections managed by the French veterinary health services (34). Similarly, accessing various datasets owned by producer organizations (economic performance and biosecurity audits) made it possible to assess risk factors of contamination by *Campylobacter* (41). Slaughterhouse databases (42, 43) and welfare control databases (44) also have proved to be extremely useful in efforts to address similar changes. In such cases, it is a win-win situation, where database owners may profit from data they collected for purposes others than research and that they did not plan to analyze themselves. Finally, concerning database analysis, a crucial aspect involves how feedback is provided, which could be at the farmer level or more widely to a broader audience. The farmer level feedback reported in this study mostly involved personalized reports. This is not surprising since certification, advice, and enforcement of regulations aim to present results directly to the farmer, with the ultimate objective being to improve biosecurity compliance on the farm. It also constitutes a first step to propose farm-tailored advice. Research shows that to achieve change in animal health related behavior on a farm, advice must be tailored to the characteristics and needs of that farm (45–47). Interestingly, some feedback to farmers may include a benchmark that farmers can use to compare their own biosecurity practices with those of other farmers. This is also an important tool to modify behavior, as farmers may (1) be sensitive to being compared to others (social norm) and (2) require proof that they can realistically adopt biosecurity practices on a routine basis (the concept of auto-efficacy) (48, 49). For this purpose, the results of a biosecurity assessment must be directly or at least promptly entered into a digital database so that a comparison between the farmer and the general population of farmers who use that assessment tool can be made. An efficient way to present such comparisons is to produce radar plots (9). Smartphone apps and web-based tools may offer the possibility to enter the results easily and obtain feedback instantly. Feedback also may be considered at a broader level in the form of reports presenting analyzed data. These reports can be communicated to concerned farmers but also to others, and serve any stakeholder seeking to assess the overall evolution of biosecurity practices. Such reports can take the form of scientific articles or open access reports by veterinary health services.

Based on all of these challenges and opportunities related to the creation, maintenance, and use of biosecurity databases, we have developed a set of recommendations. First, establishing an atlas of biosecurity databases would help all stakeholders have an overview of when, where and under what conditions biosecurity is assessed on poultry farms. This would be crucial to avoid unnecessary biosecurity assessments and would also create opportunities for data analysis by researchers. The same concept has been applied to veterinary antibiotic stewardship at the European level (50). Second, we suggest that standardizing some aspects of biosecurity assessment would help to avoid missing key biosecurity items and to exploit databases more easily and efficiently (feedback, specific reports, transversal reports, and provision of knowledge on the effects of biosecurity). This is one of the objectives behind the deployment of a standardized biosecurity assessment tool which now takes into account the specificities of different poultry production types (51). Even if all stakeholders do not agree on using the same assessment tool, a checklist of essential biosecurity items could be established and disseminated. Finally, we suggest that some meta-data should be collected on farm types, with respect to the European Union General Data Protection Regulation. This meta-data would include, for example, farmed species (boiler/layer chicken, turkey, waterfowl, etc.), production type (intensive, free-range), farm size (in terms of barn capacity or annual production), and whether farms market their products through short supply chains.

While this study was conducted in seven countries and only in the poultry industry, the results presented here and the general concepts behind the use of biosecurity databases may be transposed to other countries or farmed species for which the control of biosecurity is of major importance. In some countries, databases on biosecurity implementation are still absent and the organizations in charge of implementing disease control strategies may take these results into consideration.

In conclusion, although they are often related to the enforcement of biosecurity regulations, biosecurity assessment systems, and their databases form a heterogeneous whole. Biosecurity assessments may have multiple and sometimes unexpected objectives. Efforts to identify existing biosecurity assessment systems and to increase the accessibility of databases related to these systems need to continue. The databases also should be analyzed more frequently in order to provide high quality feedback to both farmers and other stakeholders, and to produce more evidence on the benefits of biosecurity. Listing biosecurity databases and analyzing their results on a broader scale should help identify and share supporting measures aimed at improving biosecurity compliance.

## Data availability statement

The datasets presented in this article are not readily available because access to the dataset requires the authorization of NetPoulSafe consortium members involved in data collection. Requests to access the datasets should be directed to mattias.delpont@envt.fr.

## Author contributions

MD, MP, J-LG, AZ, A-CD-L, and JD contributed to the conception and the design of the study. MD, JD, AZ, PS, A-CD-L, NR, AS, AA, GT, AP, AD, SS-N, HM, LK, and ÁJ collected the data. MD and LS organized the database and performed the analysis. MD and MP wrote the first draft of the manuscript. All authors contributed to the article and approved the submitted version.

## Funding

This project has received funding from the European Union’s Horizon 2020 research and innovation program under grant agreement No. 101000728 (NetPoulSafe). The results presented reflect the authors’ view. The Agency is not responsible for any use that may be made of the information it contains.

## Conflict of interest

The authors declare that the research was conducted in the absence of any commercial or financial relationships that could be construed as a potential conflict of interest.

## Publisher’s note

All claims expressed in this article are solely those of the authors and do not necessarily represent those of their affiliated organizations, or those of the publisher, the editors and the reviewers. Any product that may be evaluated in this article, or claim that may be made by its manufacturer, is not guaranteed or endorsed by the publisher.
