# Supplementary material for: Monitoring biosecurity in poultry production: an overview of databases reporting biosecurity compliance from seven European countries
Source: Front Vet Sci. 2023 Aug 15;10:1231377. doi: 10.3389/fvets.2023.1231377 (PMC10465163; doi:10.3389/fvets.2023.1231377)
Supplement: Supplementary file 1 [file Data_Sheet_1.docx]

Supplementary Material

Monitoring biosecurity in poultry production: an overview of databases reporting biosecurity compliance from seven European countries

Mattias Delpont^1*^, Luis G Salazar^1^, Jeroen Dewulf ^2^, Artur Zbikowski^3^, Piotr Szeleszczuk^3^, Anne-Christine Dufay-Lefort^4^, Nathalie Rousset^4^, Annick Spaans^5^, Arthi Amalraj^2^, Giuditta Tilli^6^, Alessandra Piccirillo^6^, Aitor Devesa^7^, Sandra Sevilla-Navarro^7^, Hilde van Meirhaege^8^, László Kovács^9^, Ákos Bernard Jóźwiak^10^, Jean-Luc Guérin^1^, Mathilde C Paul^1^

*** Correspondence:** Corresponding Author: mattias.delpont@envt.fr

# Supplementary Figures and Tables

For more information on Supplementary Material and for details on the different file types accepted, please see [here](https://www.frontiersin.org/guidelines/author-guidelines#supplementary-material).

## Supplementary Tables

**
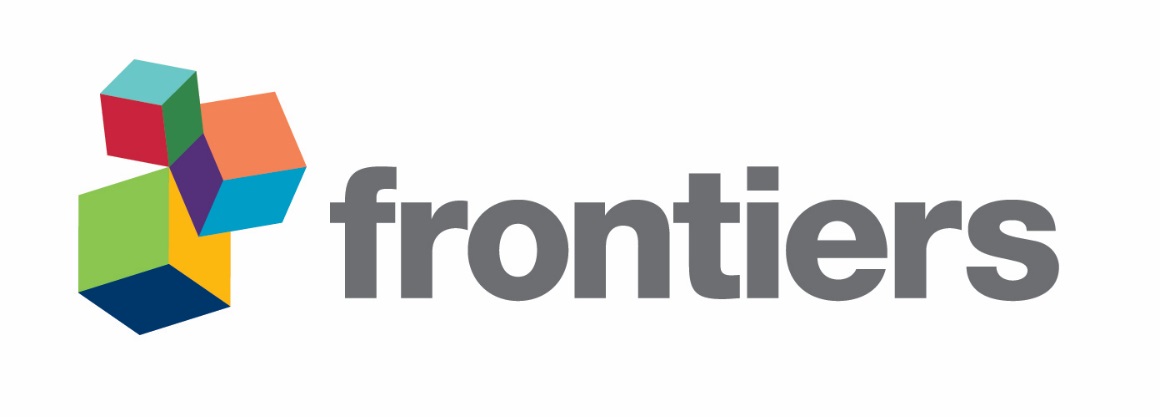
**

**Supplementary Table 1.** Questionnaire used to describe the databases informing on biosecurity in poultry farms in the seven participating countries..

| **Part 1. Context of the databases** | |  |
| --- | --- | --- |
| In which country was the data collected (you may choose more than one country)? | |  |
|  | Belgium |  |
|  | France |  |
|  | Hungary |  |
|  | Italy |  |
|  | Poland |  |
|  | Spain |  |
|  | The Netherlands |  |
| How is structured the database? | |  |
|  | One or few digital files |  |
|  | On sheets of paper |  |
|  | Multiple digital files, one for each farm/hatchery |  |
|  | Other |  |
| Please give more details (how is structured the database) | |  |
|  | (free text) |  |
| Can you give or estimate the number of farms/hatcheries which are concerned | |  |
|  | Yes |  |
|  | No |  |
| How many farms/hatcheries are concerned | |  |
|  | (free text) |  |
| What is the origin of the data | |  |
|  | Official veterinary authorities |  |
|  | Public institution |  |
|  | Producer organisation (integration, slaughterhouse, processing plant) |  |
|  | Market |  |
|  | Non-governmental organisation |  |
|  | Interprofessional organisation |  |
|  | Research institute, university |  |
|  | Quality label |  |
|  | Veterinary organisation |  |
|  | Feed or equipment supplier |  |
|  | Pharmaceutical or hygiene company |  |
|  | Advisor |  |
|  | Other |  |
| If you ticked "other", please give more details (what is the origin of the data) | |  |
| Comments |  |  |
|  | (free text) |  |
| Is the data publicly available? | |  |
|  | Yes |  |
|  | No |  |
| Is the data available upon request? | |  |
|  | Yes |  |
|  | Yes, under certain conditions |  |
|  | No |  |
| Under which conditions could the data be used? | |  |
|  | (free text) |  |
| Could you give your reasons for not wanting to share the data | |  |
|  | (free text) |  |
| Who owns the data? |  |  |
|  | (free text) |  |
| Please provide more details or comments on data ownership | |  |
|  | (free text) |  |
| What was the aim of data collection? | |  |
|  | Provide a certification (e.g., quality scheme) |  |
|  | Provide an inventory |  |
|  | Enforce compliance with national regulations |  |
|  | Provide advice |  |
|  | Research |  |
|  | Condition for a (financial) incentive |  |
|  | Other |  |
| If other, please detail (what was the aim of data collection) | |  |
|  | (free text) |  |
| Any comment on the aim of data collection? | |  |
|  | (free text) |  |
| Which species/productions are concerned? | |  |
|  | Broiler - indoors |  |
|  | Broiler - free range |  |
|  | Turkey - indoors |  |
|  | Turkey - free range |  |
|  | Duck - indoors |  |
|  | Duck - free range or foie gras |  |
|  | Egg-layer - indoors |  |
|  | Egg-layer - free range |  |
|  | Other |  |
| For other, specify (which species/productions are concerned) | |  |
| Which production stages are concerned? | |  |
|  | Selection or multiplication (breeders) |  |
|  | Hatchery |  |
|  | Final Production |  |
|  | Don't know |  |
| Do you know the time span of the biosecurity data? | |  |
|  | Yes |  |
|  | No |  |
| Data on biosecurity: beginning date | |  |
|  | (choose a date) |  |
| Data on biosecurity: ending date | |  |
|  | (choose a date) |  |
| If you don't know precisely the time span of the biosecurity data, can you provide the information you do have? | | |
|  | (free text) |  |
| How "old" is the biosecurity data collection system (years)? You may give an estimation. | |  |
|  | (free text) |  |
| How was biosecurity assessed? | |  |
|  | Postal questionnaire |  |
|  | Online questionnaire |  |
|  | Telephone interview |  |
|  | Face-to-face interview |  |
|  | Farm visit |  |
|  | Other |  |
| If other, please precise (how was biosecurity assessed) | |  |
|  | (free text) |  |
| Do you wish to add some comments? | |  |
|  | (free text) |  |
| Who assessed biosecurity? | |  |
|  | Researcher |  |
|  | Technician |  |
|  | Farmer/owner |  |
|  | Farm vet |  |
|  | External auditor (e.g., quality scheme) |  |
|  | Public health authority vet or technician |  |
|  | Other |  |
| If you ticket "other", please specify (who assessed biosecurity) | |  |
| Sampling: how were the farmers/farms chosen for data collection? | |  |
|  | (free text) |  |
| Coverage: could you roughly estimate the proportion of farms/hatcheries obtained in the target population (percentage)? | | |
|  | No |  |
|  | Yes |  |
| Your estimation |  |  |
|  | (free text) |  |
| Compulsory: were the farms/hatcheries forced to participate? | |  |
|  | Yes |  |
|  | No |  |
|  | I do not know / I am not sure |  |
| Feedback: are the results of data collection given to the farmers/hatcheries? | |  |
|  | Yes |  |
|  | No |  |
|  | I do not know / I am not sure |  |
| Could you give some more precision in the way the feedback were conducted? | |  |
|  | (free text) |  |
|  |  |  |
| **Part 2. Content of the databases** | |  |
| Farm characteristics (number of poultry barns or farm sites) | |  |
|  | Yes |  |
|  | No |  |
|  | I do not know / I am not sure |  |
| Flock management system: All-in/all-out | |  |
|  | Yes |  |
|  | No |  |
|  | I do not know / I am not sure |  |
| Farm site and restriction zones delimitation (fencing, gate, plan, warning signs) | |  |
|  | Yes |  |
|  | No |  |
|  | I do not know / I am not sure |  |
| Comments |  |  |
|  | (free text) |  |
| Vehicle decontamination procedures | |  |
|  | Yes |  |
|  | No |  |
|  | I do not know / I am not sure |  |
| Vehicle flow (circuit, parking) | |  |
|  | Yes |  |
|  | No |  |
|  | I do not know / I am not sure |  |
| Equipment sharing and decontamination | |  |
|  | Yes |  |
|  | No |  |
|  | I do not know / I am not sure |  |
| Comments |  |  |
|  | (free text) |  |
| Carcass management procedures (picking, storage, destination) | |  |
|  | Yes |  |
|  | No |  |
|  | I do not know / I am not sure |  |
| Droppings management procedures (retrieval, storage, evacuation) | |  |
|  | Yes |  |
|  | No |  |
|  | I do not know / I am not sure |  |
| Egg-related protocols (sanitation, handling, transport) | |  |
|  | Yes |  |
|  | No |  |
|  | I do not know / I am not sure |  |
| Farmer and staff (connection with other farms, formations in biosecurity) | |  |
|  | Yes |  |
|  | No |  |
|  | I do not know / I am not sure |  |
| Visitors (restrictions, log) |  |  |
|  | Yes |  |
|  | No |  |
|  | I do not know / I am not sure |  |
| Hygiene locks / Anterooms (number, associated equipment and practices) | |  |
|  | Yes |  |
|  | No |  |
|  | I do not know / I am not sure |  |
| Poultry flow procedures (unloading, loading, within-farm movements) | |  |
|  | Yes |  |
|  | No |  |
|  | I do not know / I am not sure |  |
| Vermin management procedures, including rodents and insects (clear areas, baiting, feed storage conditions) | | |
|  | Yes |  |
|  | No |  |
|  | I do not know / I am not sure |  |
| Wild birds (bird-proofing) |  |  |
|  | Yes |  |
|  | No |  |
|  | I do not know / I am not sure |  |
| Comments |  |  |
|  | (free text) |  |
| Water (origin, sanitation) |  |  |
|  | Yes |  |
|  | No |  |
|  | I do not know / I am not sure |  |
| Bedding (type, origin, storage conditions, handling procedures) | |  |
|  | Yes |  |
|  | No |  |
|  | I do not know / I am not sure |  |
| Comments |  |  |
|  | (free text) |  |
| Cleaning protocols (for poultry barns, including feeders/drinkers and air inlets/outlets) | |  |
|  | Yes |  |
|  | No |  |
|  | I do not know / I am not sure |  |
| Disinfection protocols |  |  |
|  | Yes |  |
|  | No |  |
|  | I do not know / I am not sure |  |
| Downtime duration |  |  |
|  | Yes |  |
|  | No |  |
|  | I do not know / I am not sure |  |
| Farm cleanability (concrete or cobbled zone at barn entrance, type of flooring, wall and roof cleanability, house age) | | |
|  | Yes |  |
|  | No |  |
|  | I do not know / I am not sure |  |
| Comments |  |  |
|  | (free text) |  |
